# Anti-Leptospira Seroprevalence and Associated Risk Factors among Forestry Workers in Lower Saxony, North-West Germany

**DOI:** 10.3390/microorganisms12071262

**Published:** 2024-06-21

**Authors:** Christiane M. Klier, Christina Princk, Martin H. Richter, Enno Luge, Anne Mayer-Scholl, Maren Mylius, Kristin Maria Meyer-Schlinkmann, Sophie Rettenbacher-Riefler, Masyar Monazahian, Armin Baillot, Rainer G. Ulrich, Johannes Dreesman

**Affiliations:** 1 Public Health Agency of Lower Saxony, 30449 Hannover, Germany; christina.princk@gmx.de (C.P.); maren-mylius-pub@gmx.net (M.M.); sophie.rettenbacher-riefler@nlga.niedersachsen.de (S.R.-R.); masyar.monazahian@nlga.niedersachsen.de (M.M.); armin.baillot@nlga.niedersachsen.de (A.B.); johannes.dreesman@nlga.niedersachsen.de (J.D.); 2 Department of Biological Safety, German Federal Institute for Risk Assessment, 10589 Berlin, Germany; martin.richter@bfr.bund.de (M.H.R.); enno.luge@bfr.bund.de (E.L.); anne.mayer-scholl@bfr.bund.de (A.M.-S.); 3 Institute of Novel and Emerging Infectious Diseases, Friedrich-Loeffler-Institut, Federal Research Institute for Animal Health, 17493 Greifswald-Insel Riems, Germany; rainer.ulrich@fli.de

**Keywords:** forestry workers, leptospirosis, Germany, seroprevalence, risk factors, re-emerging, rodent-borne disease

## Abstract

As leptospirosis is re-emerging, a seroprevalence study was conducted, assessing the prevalence of anti-Leptospira IgG antibodies and infection-associated risk factors among forestry workers (FWs) in Lower Saxony, Germany, to develop targeted public health measures. Sera of 877 FWs, sampled in 2016, were tested for anti-Leptospira seropositivity by commercial IgG-ELISA. Data on demographics and Leptospira-specific exposures, knowledge, sources of information, and preventive measures were collected by standardized, self-administered questionnaire. A subset of 244 sera was retested via in-house IgG-ELISA. Risk factors were assessed from the subset using multivariable logistic regression analysis. The commercial IgG-ELISA revealed a seroprevalence of 4.8% (95% confidence interval CI95 = 3.5–6.4). Of the 601 FWs who completed the questionnaire, 67.9% had been informed about leptospirosis and *Leptospira* spp., mainly by employers (55.2%) and peers (38.9%). Positive associations with seropositivity were observed for canoeing (adjusted odds ratio (aOR) = 2.35, *p* = 0.044), touching rodents (aOR = 2.4, *p* = 0.021), and living close to beech trees (aOR = 2.18, *p* = 0.075). Frequently cleaning animal stables was negatively associated (aOR = 0.20, *p* = 0.002). The unexpected positive association with wearing gloves when handling plants and soil (aOR = 2.16, *p* = 0.011) needs further discussion. Overall, seroprevalence was in the range of other studies in Germany. The identified factors will be used to develop targeted information reaching out to at-risk groups tapping various communication channels.

## 1. Introduction

Leptospirosis is a re-emerging rodent-borne zoonotic disease with worldwide abundance characterized by mild to severe clinical manifestations with sometimes fatal outcomes. Its etiologic agents are spirochetes of the genus *Leptospira*. The disease’s (re-)emergence is thought to be fostered by climate change and the accompanying increase in suitable environmental conditions, including food availability and the subsequent abundance of the reservoir hosts of these pathogens, as well as flooding events [1,2,3,4]. In addition, leptospirosis is considered a One Health issue for humans and animals, fostered by climate change and changes in human–animal–environmental interactions. Transmission of *Leptospira* spp. to humans occurs through direct contact with infected rodents and other mammals. However, the indirect transmission through contact with *Leptospira*-contaminated wet soils or waterbodies is the major pathway for infection, the latter being particularly closely related to flooding events. Although abundant in the tropical and subtropical regions due to climate change associated with severe weather events (e.g., heavy rains, storms, and flooding), leptospirosis is becoming a (re-)emerging public health issue in regions of temperate climate, affecting human and animal welfare [5,6,7].

Infectious bacteria are excreted in the urine of reservoir and carrier mammals such as rodents, dogs, or livestock and remain infective in the environment [3,5]. Human *Leptospira* infection results from exposure to infectious urine via the mucosa or broken skin, either directly or indirectly, in contaminated soil or water, for example, during leisure/recreational or occupational activities [8,9]. The clinical symptoms range from mild, influenza-like symptoms to severe infections with renal and hepatic failure, pulmonary distress, or even death [1,10]. The occupational at-risk groups for leptospirosis are farmers, harvest workers, veterinarians, and abattoir workers [6,10,11].

Since 2001, laboratory confirmation of an acute *Leptospira* infection is subject to mandatory reporting under the Infection Protection Act (IfSG) in Germany. From 2001–2022, the annual incidence of reported cases ranged from 0.04/10^5^ (2016) to 0.75/10^5^ (2014) population [12]. Within Europe (EU/EEA (European Economic Area)), from 2007 to 2022, the annual reported incidence ranged from 0.09/10^5^ (2013) to 0.22/10^5^ (2007), 20% of which were travel-associated cases [13].

In Germany, leptospirosis was endemic among harvest workers until the 1960s [14,15,16]. Nowadays, leptospirosis is contracted in the course of recreational activities such as triathlon, swimming, canoeing, or river surfing [8,17,18,19,20,21,22]. However, in 2007 and 2014, leptospirosis outbreaks were reported among strawberry harvesters in two different regions of Germany [23,24,25]. After leptospirosis outbreaks occurred in the aftermath of flooding events in several European countries in June 2021, the European Centre for Disease Prevention and Control (ECDC) released a risk assessment [26].

Occupational risks are also reported for forestry workers (FWs). However, information on Leptospira seroprevalence among this occupational group is scarce [27]. A Leptospira seropositivity of 14.2% was reported for FWs in North Rhine-Westphalia [28]. In a population-based cross-sectional zoonosis survey in Baden-Wuerttemberg, an overall Leptospira seroprevalence of 4.2% and a relative risk of 9.2 of Leptospira seropositivity among FWs compared to non-forestry workers were reported [21].

### Aim of the Study

This study aimed at assessing the anti-Leptospira immunoglobulin G (IgG) antibody prevalence and factors associated with Leptospira seropositivity among an occupational at-risk group of FWs The identified factors can be used to develop targeted materials raising awareness of leptospirosis, its etiologic agent, and preventive measures related to both recreational and occupational activities.

## 2. Material and Methods

### 2.1. Participants and Study Design

The study population comprised employees of the three forestry services in Lower Saxony, namely Lower Saxony State Forest, Northwest German Forest Research Institute, and Harz National Park Service. Participation was voluntary and no incentives were given.

**Figure 1:**
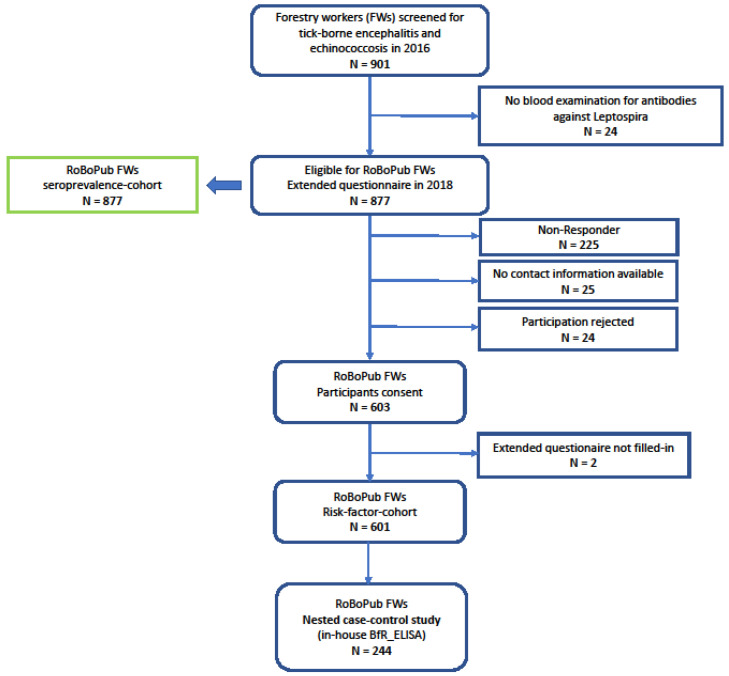
Flow chart of forestry worker cohort. RoBoPub: Rodent-Borne-Pathogens-and-Public-Health; FWs: forestry workers.

In the course of a biannual serum panel on “Surveillance of Tick-borne encephalitis and Echinococcosis” among forestry workers (FWs), the Public Health Agency of Lower Saxony (NLGA) offered testing on anti-Leptospira IgG to participating FWs within the 2016 round of surveillance. The panel’s protocol comprised serum sampling, a short questionnaire, and written consent. The sera were screened for anti-Leptospira IgG, and the test results were communicated to the FWs in 2017. In 2018, the FWs of the 2016 round were invited to fill in an extended paper-based, standardized questionnaire addressing potential risk factors associated with anti-Leptospira seropositivity (Figure 1).

The questionnaire included baseline demographic questions, including duration of school attendance and smoker status (smoking is regarded as a risk factor for many infectious diseases, e.g., hantavirus infection) [24]. The major part of the questionnaire collected information on potential Leptospira-specific risk factors, such as exposure related to residence, occupation, occupational activity, and recreational activities, but also on adherence to preventive measures and knowledge about the pathogen, infection-related symptoms, and resources to acquire information (see Supplementary Material). The questions were derived from previous studies [19,21,28,29].

### 2.2. Laboratory Investigations

Serum samples were tested for the presence of anti-Leptospira IgG by *Serion ELISA classic* and results were scored as positive, negative, or borderline, according to the manufacturer’s recommendations (Virion, Würzburg, Germany). According to the manufacturer, the ELISA had a sensitivity and specificity of 94.7% and 99%, respectively, for both healthy blood donors and patients with suspected leptospirosis in Germany.

A subsample of participating FWs who had given written consent for additional testing of serum samples comprising all seropositive, all borderline, and a subset of randomly selected seronegative samples were subjected to an IgG in-house ELISA at the Consultant Laboratory for Leptospira at the German Federal Institute for Risk Assessment (BfR) [25]. In the case of sub-clinical infections, the sensitivity and specificity of this IgG-ELISA are 85.7% and 99.1%, respectively [30].

### 2.3. Case Definitions

Anti-Leptospira serostatus was categorized by applying two seropositivity case definitions:

1. NLGA_pos, NLGA_bl, and NLGA_neg refer to a positive, borderline, or negative screening test result, respectively, obtained at NLGA by *Serion ELISA classic*, further denoted as NLGA_ELISA.
2. BfR_pos and BfR_neg refer to a positive or negative test result, respectively, obtained by BfR anti-IgG in-house ELISA, further denoted as BfR_ELISA.

In the statistical analysis of associations with risk factors, which is described below, borderline test results of the NLGA_ELISA were categorized as negative in order to obtain a binary outcome variable.

### 2.4. Estimation of Seroprevalence

NLGA_ELISA was applied to all sera. Therefore, a seroprevalence can be calculated directly for this test. BfR_ELISA was applied to all sera for which consent was given and that were either NLGA_pos or NLGA_bl, but it was only applied to a random subsample of NLGA_neg sera. Therefore, the latter are underrepresented in the BfR_ELISA sample, and, thus, Leptospira seroprevalence cannot be calculated directly from this subsample.

In order to achieve an estimate of the seroprevalence with respect to BfR_ELISA for the full sample, for each of the three NLGA_ELISA outcomes, the proportion of BfR_pos was calculated. These proportions were multiplied with the total number in the respective category of the full sample. The results were summarized across categories to obtain the total predicted number of positives. This predicted total number of positives was divided by the size of the full sample to obtain the estimate.

### 2.5. Analysis of Risk Factors and Data Management

We assumed that the BfR_ELISA has a higher validity than the NLGA_ELISA because, e.g., it covers a broader range of *Leptospira* serotypes. Therefore, we used the BfR_pos case definition as outcome for the epidemiological analysis of risk factors. We conducted this analysis according to a nested case control study design which provides an appropriate framework for this situation [31].

We used univariable and multivariable logistic regression analysis to assess associations of anti-Leptospira seropositivity with risk factors. Differences between BfR_pos (cases) and BfR_neg (controls) were assessed by Wald test (univariable logistic regression) with 2-sided *p* values. Multicollinearity between exposures was assessed via correlation analysis; the cut-off was set at |r| > 0.3. For further analysis, one of the correlated variables was kept as proxy and the others excluded. Independent risk factors associated with BfR_pos were included in the multivariable analysis by stepwise backward selection, including variables with *p* ≤ 0.25 in the univariable analysis. The independent variables “sex” and “age” were included as forced-in variable. Adjusted odds ratios (aOR) are reported.

Age groups were categorized according to median and quartiles. “Tending to occupational outdoor activities” was coded according to time spent indoors/office and outdoors, respectively, (0: ≥ 50; office, 1: > 50 outdoor activities, such as inspecting forest or tending to active work e.g., lumbering).

Due to the low number of participants of two out of the three services, calculation of the differences between the services was not pursued.

The level of statistical significance was set to 0.05.

Both data from questionnaire and laboratory testing were filed and stored in MS-ACCESS^®^. All statistical analyses were performed using Stata 17^®^ (StataCorp LLC., College Station, TX, USA).

### 2.6. Ethics Approval

Written informed consent was obtained from each participant. The study was approved by the Ethics Committee of the General Medical Council of Lower Saxony (Sign Bo/11/2006, Bo/13/2018).

## 3. Results

### 3.1. Baseline Data—Complete Sample with Short Questionnaire (n = 877)

In 2016, 877 forestry workers (FWs) (89.5% of whom were male) provided a blood sample and completed the short self-administered questionnaire. The participating FWs were employees of Lower Saxony State Forest (N = 823, 93.8%), Northwest German Forest Research Institute (N = 20; 2.3%), and Harz National Park Service (N = 33; 3.8%); one forestry worker did not answer this question. The median age of the FWs was 50 years, ranging from 16 to 65 years (females: 49 yrs, range: 17–64 yrs; males: 51 yrs, range: 16–65 yrs). A total of 238 (37.4%) of the FWs tended primarily to indoor activities (≥50% of working time) (Table 1).

Among this cohort, 42 (4.8%; 95%-CI 3.5–6.4) tested NLGA_pos, 21 (2.4%) NLGA_bl, and 814 NLGA_neg.

None of the FWs reported having received either a clinical or a laboratory-confirmed diagnosis of leptospirosis prior to enrolment in the 2016 study.

Although the proportion of NLGA_pos in the older age group of FWs was almost twofold higher compared to the lower age group (age ≤ 50 yrs) of FWs, this association was not significant. Other associations with the basic demographic characteristics were not observed (Table 1).

### 3.2. Baseline Data—Subset with Extended Questionnaire (n = 601)

In 2018, a subset of 601 FWs completed the extended self-administered questionnaire, corresponding to a response rate of 68.1% (flow chart in Figure 1). This cohort of 601 comprised 89.9% male respondents. Since the blood collection in 2016, 35 (5.8%) respondents reported having retired.

The median age was 53 yrs, ranging from 18 to 67 years (females: 50 yrs, range: 18–65 yrs; males: 54 yrs, range: 18–67 yrs) at the time of the questionnaire. Overall, 32.8% (197/601) tended primarily to indoor activities (≥50%) (Table 1). A total of 296 (49%) had received higher education, holding secondary school certificates received for completing school higher than grade 10. The median duration of employment was 31 yrs (range: 2–50 yrs).

A history of smoking either in the present or in the past was reported by 264 FWs (44%) with a mean duration of 22.8 yrs (range: 1–50 yrs). Of the 105 FWs who still smoked, a median smoking duration of 35 yrs (1–50 yrs) was reported; FWs who quit smoking reported a mean smoking history of 15 yrs (n = 157). Among this cohort of FWs, 310 (51.6%) reported being nonsmokers.

Overall, 408 (67.9%) FWs reported having knowledge of *Leptospira* spp. and leptospirosis. The major source of information mentioned was the employer (332; 55.2%); followed by friends, peers, and relatives (234; 38.9%); and local public health authorities (196; 32.6%). Traditional media such as print media or television/radio were mentioned by 169 (28.1%) and 136 (22.6%) respondents, respectively. Only 89 (14.8%) FWs reported retrieving information via social media or the internet. Analysis of the clinical symptoms associated with anti-Leptospira seropositivity was not pursued since participants had filled in the questionnaire after they had been informed about the serological results. We expected the answers to be biased.

Among this subset of 601 FWs, 27 (4.5%) tested NLGA_pos (24 (4.5%) out of the 537 males and 3 (4.7%) out of the 64 females), and 20 FWs tested NLGA_bl. (Table 1).

None of the factors “age”, “sex”, and “smoking” were significantly associated with NLGA_pos (Table 1). This also applied to “duration of service” (N = 523; *p* = 0.747).

“Knowledge of *Leptospira* spp. and leptospirosis” was significantly higher among NLGA_pos FWs (OR: 4.0, *p* = 0.026) in comparison to all other FWs.

### 3.3. Anti-Leptospira Serostatus and Associated Risk-Factors—Nested Case Control Study (N = 244)

#### 3.3.1. Serological Results and Anti-Leptospira Serostatus

A subsample of 244 sera comprising 25 NLGA_pos, 20 NLGA_bl, and 199 NLGA_neg (random selection) sera was subjected to retesting at BfR. All of the sera which were NLGA_pos tested positive in BfR_ELISA, but only 65% of those initially tested NLGA_bl. Of those sera initially tested NLGA_neg, 5.5% tested positive in BfR_ELISA. If these rates were applied to the initial full sample of 877, we would expect 100 BfR_ELISA positive results, corresponding to a seroprevalence of 11.4%, compared to the NLGA_ELISA seroprevalence of 4.8% (Table 1 and Table 2).

In total, of the 244 FWs whose sera were tested with BfR_ELISA, 49 tested BfR_pos and 195 BfR_neg. In the following, the former will be denoted as cases and the latter as controls.

#### 3.3.2. Analysis of Risk Factors Associated with Anti-Leptospira Serostatus

There were no significant differences between cases and controls related to either sex (aOR of females: 0.62, *p* = 0.685) or age distributions (aOR: 1.06 of higher age group compared to younger age group, *p* = 0.917) (Table 3).

Cases had 1.6 times increased odds of tending to outdoor activities in the univariable analysis, which was not confirmed by multivariable analysis (Table 3). Cases had 48% reduced odds of holding a higher school certificate (>grade 10) (OR: 0.52; *p* = 0.091) in the univariate analysis and 57% increased odds of being smokers (OR:1.57, *p* = 0.254; Table 3).

In the multivariable analysis, cases had increased odds of touching rodents (aOR: 2.4, *p* = 0.021), canoeing (aOR: 2.4, *p* = 0.044), or consciously wearing gloves when handling plants and soil (aOR: 2.16, *p* = 0.011) (Table 3). Although living close to beech trees was associated with a more than 2-fold increase in odds, it was not significant (aOR: 2.2, *p* = 0.075). Cases had 75% reduced odds of frequently cleaning animal stables or animal cages (aOR: 0.35, *p* = 0.002) and 51% reduced odds of using a garage (aOR: 0.49; *p* = 0.075) (Table 3). Frequent swimming was associated with increased odds of seropositivity in the univariable analysis (OR; 2.4, *p* = 0.043) (Table 3); due to collinearity with canoeing, frequent swimming was dropped in the multivariate analysis.

## 4. Discussion

We report the Leptospira seroprevalence and factors associated with Leptospira seropositivity among a cohort of FWs in Lower Saxony. The seroprevalence of anti-Leptospira IgG assessed by NLGA_ELISA (*Serion ELISA classic*) was 4.8% among FWs in Lower Saxony, which was higher than the value of 2.4% measured with NLGA_ELISA among participants of a cross-sectional study conducted in a rural area of Lower Saxony without a specific forest-associated occupation [28,32]. The predicted seropositivity of 11.4% with respect to BfR_ELISA lies in the range reported for FWs in North Rhine-Westphalia, also tested by BfR_ELISA (14.2%) [28].

For the interpretation of these results, one has to keep in mind that *Serion ELISA classic*, in contrast to BfR_ELISA, does not cover *Leptospira kirschneri serovar* Grippotyphosa and that this serotype has been associated with illness and at least one outbreak in Germany, implying that real Leptospira seropositivity among FWs may be higher [25]. Also, *Serion ELISA classic* was developed for the clinical diagnosis of patients and not seroprevalence studies in healthy individuals. In addition, a recent study in bank vole (*Myodes glareolus*) reservoirs indicated a mean prevalence of 7.5% for the pathogenic *Leptospira* species *L. interrogans*, *L. kirschneri*, and *L. borgpetersenii* in a part of Lower Saxony [33].

None of the Leptospira-seropositive FWs reported either a laboratory-confirmed or clinical diagnosis of leptospirosis. This finding of seropositive individuals not remembering a specific set of symptoms or a diagnosis was also seen in a population-based, cross-sectional study in Baden-Wuerttemberg and is in support of the observation that *Leptospira* infections rarely become obvious [21,34]. Due to nonspecific symptoms with a subclinical or mild clinical course, infections with *Leptospira* spp. often go unnoticed or are not taken into consideration. This causes underdiagnosis, resulting in underreporting via the notification system [21].

In Lower Saxony, mean annual incidence of reported cases is 0.16/10^5^ population (range during 2001–2022: 0.04–0.75/10^5^ population), corresponding to a mean 12.3 reported cases/year throughout Lower Saxony (median 9, range 3–49 cases during 2001–2022; [12]). In 2014, due to an outbreak among harvesters, incidence in Lower Saxony reached 3.3/10^5^ population [12].

The core of our analysis was the 244 samples for which data from the extended questionnaire were available and which were retested with BfR_ELISA. The BfR_ELISA confirmed all previously NLGA_ELISA-positive tested samples from the investigated subset. However, positive results were also detected by BfR_ELISA from the subsets of NLGA_bl and NLGA_neg samples (Table 2). Still, the BfR_ELISA revealed 28 seropositive samples that were previously tested negative or borderline by the NLGA_ELISA. As stated previously, BfR_ELISA detects a wider range of antibodies against pathogenic *Leptospira* species, and this may be the reason for the higher rate of positive samples in the retested sample. These findings further underline reports of underdiagnosis by commercially available anti-Leptospira IgG serological tests [21,35].

The assessment of associations with Leptospira seropositivity by means of nested case control study analysis revealed that cases had increased odds of residing in the vicinity of beech trees (aOR: 2.2, *p* = 0.075), which was similarly observed in a previous study where a 1.7-fold increased relative risk when living close to a forest was reported [21,29].

The finding that canoeing and swimming increased the risk of Leptospira seropositivity is in accordance with other studies [21,29]. There are a number of reports on outbreaks on the occasion of recreational outdoor activities, such as triathlon or kayaking, which are in support of the increased odds of Leptospira seropositivity associated with canoeing or swimming in our study [2,8,17,18,19,20,21,22]. Contact with infectious material, e.g., contaminated water or soil, can also occur during canoeing trips, either during overnight camping or in the sheds where canoes/kayaks are stored.

Contact with rodents and sighting of rodent nests increased the odds of Leptospira seropositivity (aOR: 2.4, *p* = 0.021; aOR: 2.0; *p* = 0.065). This finding was also observed in previous studies, where a 2.6-fold and 1.6-fold increase in the odds and relative risks of Leptospira seropositivity were reported, respectively [21,36].

In our study, the frequent cleaning of animal stables or cages (>20 times within past 10 years) reduced the odds of Leptospira seropositivity by 80%. This finding supports the hypothesis that frequent cleaning reduces the odds of encountering rodents (both dead and alive), rodent excrement, or contaminated water and surfaces. Having a cat did not reveal any association with seropositivity, nor did the presence of domestic, companion animals, livestock, or dogs, which had been reported as factors associated with increased odds of seropositivity before [21]. An explanation for the finding that always wearing gloves while handling plants or soil increased the odds of anti-Leptospira seropositivity might apply to those FWs who are strongly engaged in gardening. However, gardening itself was not a significant risk factor. Another reason could be that infection occurs accidentally when not paying further attention, e.g., not washing hands after the removal of soiled gloves, accidentally touching the eyes or mouth while handling plants and soil, or having a more careless attitude while wearing gloves under the impression of protection. In our study, there was no significant association between Leptospira seropositivity and smoking.

FWs who held higher secondary school certificates had 46% (grade 10) and 48% (>grade 10) lower odds of Leptospira seropositivity in comparison to participants holding school certificates received at gradation up to 9th grade, in the univariable analysis (Table 3). FWs with lower secondary school certificates often hold positions involving outdoor activities, both in the fields and in the forest, thus having a higher chance of exposure to contaminated soils, water bodies, and woods [6]. A previous study described a 9.2-fold increase in the odds of Leptospira seropositivity among FWs in Baden-Wuerttemberg [21]. Increased anti-Leptospira seropositivity among FWs, hunters, and farmers are reported from Ireland, Italy, and other European countries, respectively, indicating an increased risk among these groups [27,37,38].

The findings on knowledge and sources of information for both *Leptospira* spp. and leptospirosis may be biased since the questionnaire was filled in after the serology results had been communicated to the participants. Yet, awareness needs to be raised of the risk of contracting *Leptospira* infection, both in the course of occupational as well as recreational activities. Employers could enhance the frequency of employee training to raise the awareness of pathogens and clinical manifestations, with a major focus on preventive measures such as wearing protective gear to mitigate exposure and infection. Information can be provided by tapping into various communication channels, e.g., for in-person training or providing links to relevant materials accessible by social media or websites, e.g., released by the German Federal Institute for Occupational Safety and Health or other public health authorities.

The finding that none of the Leptospira-seropositive FWs recalled earlier clinical diagnosis of leptospirosis underlines the subclinical occurrence as well as the underdiagnosis and underreporting of this zoonotic infection. This could be counteracted by raising awareness among physicians by means of specific training focusing on the diagnosis, clinical manifestations, and therapy of zoonotic diseases. Since there are no vaccines for human use available in Germany, awareness should be raised for the ecology of the pathogens, its rodent reservoirs, and the implementation of mitigation strategies.

Retrospective questionnaire surveys usually involve the risk of bias, particularly recall bias and social desirability bias. In our study, the risk of recall bias cannot be excluded. However, the majority of the questions addressed long-term behaviors, which should also be remembered over a longer period of time, irrespective of the case status. Social desirability bias mainly occurs with sensitive questions in a personal interview setting. In our study, we used self-administered questionnaires, and the majority of questions were not very sensitive. Only the results on prevention behaviors, such as wearing gloves, might have been affected by this type of bias.

## 5. Conclusions

We conclude that we observed an anti-Leptospira seropositivity that was higher than in the general rural population but not higher than in comparable occupational groups in other regions. However, we also showed that seropositivity results are greatly affected by the ELISA used. The difference in performance might be associated with the antigen used for the test. As stated previously, BfR_ELISA detects a wider range of antibodies against pathogenic *Leptospira* species, including *Leptospira kirschneri serovar* Grippotyphosa, which has been associated with illness and at least two outbreaks in Germany. Hence, our results imply that the performance of future serological tests in the region could benefit if antigens of circulating *Leptospira* serovars are included. We discovered an association with some risk factors, some of them in line with other studies, such as rodent contact, canoeing, or swimming. The rather unexpected finding of frequent use of gloves being associated with increased odds of anti-Leptospira seropositivity should be subject to follow-up. Comparison with the number of diagnosed or notified cases shows a strong under-recognition of the infection, probably due to subclinical courses and low awareness. To raise awareness of the disease, pathogens and preventive measures, targeted information needs to be developed, emphasizing both occupational and recreational outdoor activities. Messages should emphasize avoiding contact with infectious materials, implementing rodent controls, and taking protective measures, e.g., covering broken skin, handwashing before eating, and the use of protective clothing (gloves, long-sleeved shirts and pants, and closed shoes) to reduce the risk of infection, as well as seeking medical care in the case of signs of infection. While employers are key players in the communication of work-related preventive measures, it is the public health institution’s responsibility to reach out to the public.

## Figures and Tables

**Table 1:** Basic demographic characteristics and anti-Leptospira seropositivity determined by NLGA_ELISA ^$^.

			Full Sample with Short Questionnaire					Sample with Extended Questionnaire					
Characteristic		Total	pos	%	CI 95% ^#^	neg	*p* *	Total	pos	%	CI 95%	neg	*p* *
**Total**		**877**	42	4.8	3.6–6.4	835		**601**	27	4.5	2.9–6.6	574	
**Sex**	male	785	39	5.0	3.6–6.7	746	0.468	537	24	4.5	2.9–6.6	513	0.880
	female	92	3	3.3	1.1–9.6	89		64	3	4.7	0.9–13.1	61	
**Age group** ^§^	<median	437	15	3.4	1.9–5.6	422	0.061	298	13	4.5	2.5–7.8	285	0.879
	≥median	440	27	6.1	4.1–8.8	413		303	14	4.7	2.6–7.8	289	
**Outdoor work**	≤50%	238	14	5.9	3.3–9.7	223	0.355	197	9	4.6	2.1–8.5	188	0.950
(% of work time)	>50%	639	28	4.5	3.0–6.4	611		404	18	4.5	2.7–7.0	386	
**Education** School certificate (grade completed)	<10 grade	n.a.	n.a.			n.a.		133	11	8.2	4.2–14.3	122	0.040
	10 grade	n.a.	n.a.			n.a.		172	4	2.3	0.6–5.8	168	
	>10 grade	n.a.	n.a.			n.a.		296	12	4.1	2.1–7.0	284	
**Smoking**	never	n.a.	n.a.			n.a.		317	14	4.4	2.4–7.3	303	0.924
	past/present	n.a.	n.a.			n.a.		284	13	4.6	2.5–7.7	1	

^$^ Serion ELISA classic (Virion, Würzburg, Germany); ^#^ 95% confidence interval; *p*: *p*-value; * Pearson chi2; n.a., not addressed in short questionnaire; neg: negative; pos: positive; ^§^ Median: 2016 Cohort: 50 yrs, 2018 cohort: 53 yrs.

**Table 2:** Anti-Leptospira IgG serostatus among forestry workers: Screening assay (*Serion ELISA classic*, Virion) versus BfR assay (BfR in-house ELISA, Consultant Laboratory for Leptospira).

		BfR_ELISA (In-House ELISA) ^#^						
		(A) Subset Case Control Study (244)				(B) Prediction for Full Sample (877)		
		total	BfR_neg	BfR_pos	BfR_ pos (%)	total	predicted BfR_pos	predicted BfR_pos (%)
* NLGA_ELISA (commercial)	NLGA_neg	199	188	11	5.5	814	44.7	
	NLGA_bl	20	7	13	65	21	13.7	
	NLGA_pos	25	0	25	100	42	42	
		**244**	**195**	**49**		**877**	**100.4**	**11.4**

* Screening assay: *Serion ELISA classic*; ^#^ in-house ELISA: Consultant Laboratory for Leptospira; neg: negative; bl: borderline; pos: positive.

**Table 3:** Factors associated with anti-Leptospira IgG seropositivity—univariable and multivariable logistic regression.

			Leptospira Serostatus				Univariable Analysis				Multivariable Logistic Analysis		
Variable		Total	pos	%	neg	%	OR	95% CI	*p* (Wald)	*p* (X^2^)	aOR	95% CI	*p* (Wald)
**Total**		**244**	**49**		**191**								
Sex	male	215	44	20.5	171	79.5	ref			0.684			
	female	29	5	17.2	24	82.8	0.81	0.29–2.24	0.685		0.62	0.31–1.27	0.194
Age (yrs)													
	<53	116	26	22.4	90		ref			0.387			
	≥53	128	23	18.0	105	82.0	0.76	0.40–1.42	0.388		1.06	0.35–3.25	0.917
Age (yrs) ^#^													
	<45	60	13	21,7	47	76.8	ref			0.641			
	45–52	56	13	23.2	43	76.8	1.09	0.46–2.62	0.842				
	53–58	62	10	16.1	52	83.9	0.69	0.78–1.73	0.436				
	≥59	66	13	19.7	53	80.3	0.89	0.37–2.10	0.785		not included		
**On average, what portion of your working hours do you spend on outdoor activities…?**													
Outdoors	<50%	90	14	15.6	76	84.4	ref			0.177			
	≥50%	154	35	22.7	119	77.3	1.60	0.81–3.16	0.180		dropped		
Outdoors	≤50%	81	14	17.2	65	80.2	ref			0.432			
	50–70%	47	10	21.3	37	78.7	1.29	0.52–3.20					
	≥70% (forest)	116	29	25.0	87	75.0	1.59	0.78–3.3	0.199		dropped		
**What is the higest level of education you have completed?**													
School certificate (grade completed)	<grade 10	56	16	28.6	40	71.4	ref			0.195			
	grade10	67	12	17.9	55	82.1	0.54	0.23–1.27	0.163				
	>grade10	121	21	17.4	100	82.6	0.52	0.25–1.11	0.091		dropped		
**Do you currently smoke—even if only occasionally?**													
Smoking	never	131	24	18.3	107	81.7				0.500			
	in the past	63	12	19.0	51	81.0	1.05	0.50–2.26	0.903				
	present	50	13	26.0	37	74	1.57	0.72–3.39	0.254		not included		
Smoking	no	131	24	18.3	107	81.7				0.460			
	yes	113	25	22.1	88	77.9	1.23	0.67–2.37	0.460		not included		
**How many years have you lived in the following locations?**													
Urban	never	157	35	22.3	122	77.7				0.247			
	>1 yr	87	14	16.1	73	83.9	0.67	0.34–1.32	0.249		dropped		
**Have you ever lived near…?**													
An inland body of water	never/>100 m	186	42	22.6	144	77.4				0.041			
	<100 m	58	7	12.1	51	87.9	0.47	0.20–1.11	0.086		dropped		
Barn	never/>100 m	114	28	24.6	86	75.4				0.111			
	≤100 m	130	21	16.2	109	83.8	0.59	0.314–1.11	0.104		dropped		
Beech trees	no	204	37	18.3	167	81.9				0.087			
	yes	40	12	30.0	28	70.0	1.93	0.90–4.15	0.091		2.18	0.92–5.16	0.075
**How many years have you had access to the following areas/spaces within your apartment/home or adjoining property and have made use of them?**													
Tool shed	≤10 yrs	19	6	31.6	13	68.4				0.193			
	>10 yrs	225	43	19.1	182	80.9	0.51	0.18–1.42-	0.199		dropped		
Garage	≤10 yrs	51	15	29.4	36	70.6				0.061			
	>10 yrs	193	34	17.6	159	82.4	0.51	0.25–1.04	0.064		0.49	0.22–1.07	0.075
**How often have you cleaned the following rooms during the past 10 years?**													
Cellar	never	41	6	14.6	35	85.4				0.340			
	>1 to 2 times	203	43	21.2	160	78.8	1.57	0.62–3.97	0.343		dropped		
**On average during a typical work week (Mo-Fr) in the period from spring to fall, how long do you engage in the following activities?**													
Seeds, animal feed, feritlizer, or the like	no	161	37	23.0	124	77.0				0.115			
	≥1 h/week	83	12	14.5	72	86.7	0.56	0.28–1.16	0.118		dropped		
**On average during a typical weekend (Sat-Sun) in the period from spring to fall, how long do you engage in the following activities?**													
Handling plants and soil (e.g., gardening)	<1 h/week	98	14	14.3	84	85.7				0.108			
	2–5 h/week	117	30	25.6	87	74.4	2.07	1.02–4.17	0.042				
	≥6 h/week	29	5	17.2	24	82.8	1.25	0.41–3.82	0.695		dropped		
Seeds, animal feed, fertilizer, or the like	never	174	40	23.0	134	77.0				0.182			
	<1 h/week	52	6	11.5	46	88.5	0.43	0.17–1.1	0.078		not included		
	≥1 h/week	18	3	16.7	15	83.3	0.67	0.18–2.43	0.543				
Seeds, animal feed, fertilizer, or the like	never	174	40	23.0	134	77.0				0.074			
	>0 h	70	9	12.9	61	87.1	0.49	0.23–1.08	0.078		dropped		
**In your free time, how often do you engage in the following water sports?**													
Canoeing/kayaking/rafting	never	191	34	17.8	157	82.6				0.091			
	seldom/often	53	15	28.3	38	71.7	1.82	0.90–3.68	.094		**2.35**	**1.02–5.42**	**0.044**
Swimming in inland bodies of water	Never/seldom	219	39	17.8	180	82.2				0.009			
	often	25	10	40.0	15	60.0	3.1	1.29–7.36	0.012		not included		
Swimming in inland bodies of water	never	83	18	21.7	65	78.3				0.017			
	seldom	136	21	15.4	115	84.6	0.65	0.32–1.32	0.243		**0.45**	**0.22–0.91**	**0.025**
	often	25	10	40.0	15	60.0	2.4	0.92–9.25	0.071		dropped		
**In the past 10 years, how often have you discovered signs of rodents where you live/work?**													
Rodent nests	<20 times	177	31	17.5	146	82.5				0.104			
	≥20 times	67	18	26.9	49	73.1	1.73	0.89–3.36	0.106		2.01	0.96–4.49	0.065
**During the past 10 years, how often have you…?**													
Touched rodents	not at all	151	24	15.9	127	84.1				0.030			
	yes (≥1 times)	93	25	26.9	68	73.1	1.95	1.03–3.66	0.037		**2.4**	**1.14–4.97**	**0.021**
Cleaned animal cages/stables	≤20 times	174	42	24.1	132	75.9				0.013			
	>20 times	70	7	10.0	63	90.0	0.35	0.15–0.82	0.016		**0.20**	**0.074–0.54**	**0.002**
Swept/cleaned out storage rooms, garages, attics, etc.	≤10 times	84	21	25.0	63	75.0				0.165			
	>10 times	160	28	17.5	132	82.5	0.64	0.24–1.20	0.165		dropped		
**In the past have you consciously taken preventive measures to protect yourself against infections that can be transmitted by rodents or their excrement?**													
Wearing protective gloves when engaged in activities involving plants and soil	never	40	6	15.0	34	85.0				0.057			
	seldom	150	26	17.3	124	82.7	1.19	0.45–3.11	0.726				
	always	54	17	31.5	37	68.5	2.60	0.92–7.37	0.072		**2.16**	**1.19–3.94**	**0.011**

neg: negative; pos: positive; OR: odds ratio; 95% CI: 95% confidence interval; aOR: adjusted odds ratio; Mo: Monday, Fr: Friday; Sat: Saturday; Sun: Sunday.

## Data Availability

Data available upon request.

## Supplementary Materials

The following supporting information can be downloaded at: https://www.mdpi.com/article/10.3390/microorganisms12071262/s1, Questionnaire (German).
