# Supplementary material for: Anti-Leptospira Seroprevalence and Associated Risk Factors among Forestry Workers in Lower Saxony, North-West Germany
Source: Microorganisms. 2024 Jun 21;12(7):1262. doi: 10.3390/microorganisms12071262 (PMC11279034; doi:10.3390/microorganisms12071262)
Supplement: Supplementary file 1 [file microorganisms-12-01262-s001.zip › microorganisms-3062232-supplementary.pdf]

Teilprojekt 1

Niedersächsisches Landesgesundheitsamt

ID-Nr.

Befragung von Forstbediensteten

in Niedersachsen

Im Rahmen eines Verbundprojektes* zur „Verbesserung der Öffentlichen Gesundheit durch ein besseres Verständnis der Epidemiologie nagetierübertragener Krankheiten“

Niedersächsisches Landesgesundheitsamt (NLGA)
Roesebeckstr. 4-6
30449 Hannover

Ihre Ansprechpartnerinnen im NLGA:
Kristin Schlinkmann Tel.: 0511-4505-266
Christina Princk Tel.: 0511-4505-268

Gefördert vom:
Bundesministerium für Bildung und Forschung

* Verbundprojekt RoBoPub: Rodent Borne Pathogens and Public Health; deutsche Übersetzung: Nagetierübertragene Erreger und öffentliche Gesundheit

Liebe Studienteilnehmerin, lieber Studienteilnehmer,

Vielen Dank, dass Sie sich für eine Teilnahme an unserer Studie entschieden haben. Sie leisten damit einen wertvollen Beitrag in der Erforschung von Hantavirus-Erkrankungen und Leptospirosen.

Die Beantwortung des Fragebogens wird **ca. 20 Minuten** Ihrer Zeit in Anspruch nehmen. Nähere Informationen zum Ziel der Studie und zum Umgang mit Ihren Daten entnehmen Sie bitte der beiliegenden Studieninformation.

Sollten noch Fragen offen geblieben sein, kontaktieren Sie uns gerne unter den angegebenen Kontaktdaten.

Hinweise zum Ausfüllen des Fragebogens

- Lesen Sie jede Frage vor dem Beantworten sorgfältig durch und markieren Sie dann die Antwort, die am besten auf Sie zutrifft. Kreuzen Sie gegebenenfalls auch „Nein" oder „Weiß nicht" als Antwort an, wenn diese Antwort am besten zutrifft.
- In die folgenden Felder tragen Sie bitte Zahlen ein.

Beispiel: **Ausfülldatum des Fragebogens:** |1|4|.|0|6|.|2|0|1|8|

- Bei der folgenden Art von Fragen entscheiden Sie sich bitte zwischen den Antwortvorgaben und kreuzen Sie das für Sie zutreffende an.

Beispiel: **Geschlecht:** ☒ Männlich ☐ Weiblich

- Bei einigen Fragen haben Sie die Möglichkeit, eine Antwort in eigenen Worten zu formulieren. Nutzen Sie bitte, die dafür vorgesehenen Linien und schreiben Sie nach Möglichkeit in Blockschrift oder Druckbuchstaben.

Beispiel: **In welchem Landkreis bzw. in welcher kreisfreien Stadt wohnen Sie aktuell und seit wann?**

LANDKREIS OLDENBURG seit: 2008 oder 10 Jahren

- Falls Sie eine Antwort korrigieren möchten machen Sie dies bitte genau kenntlich.

Beispiel:

**15. Wie häufig unternehmen Sie in Ihrer Freizeit folgende Aktivitäten „im Grünen"?**			
	Nie	Selten	Häufig
spazieren gehen, wandern, joggen	☒ (korrigiert)	☒	☐

Herzlichen Dank!
Ihr NLGA-Team

FRAGEBOGEN

Ausfülldatum des Fragebogens:

I__I__I.I__I__I.I__I__I__I__I

Allgemeine Personenangaben

Geschlecht: ☐ Männlich

☐ Weiblich

Alter: I__I__I Jahre

In welchem Landkreis bzw. in welcher kreisfreien Stadt wohnen Sie aktuell und seit wann?

____ seit: ________________________

In welchem Landkreis bzw. in welcher kreisfreien Stadt haben Sie die längste Zeit in Ihrem Leben gewohnt?

____

Angaben zur Wohnlage

1. Wie viele Jahre Ihres Lebens haben Sie in folgenden Wohnlagen gewohnt?

	Nie	< 1 Jahr	2-5 Jahre	5-10 Jahre	Mehr als 10 Jahre	Ein Leben lang
In der Innenstadt	☐	☐	☐	☐	☐	☐
Am Stadtrand	☐	☐	☐	☐	☐	☐
Dörflich/ ländlich	☐	☐	☐	☐	☐	☐
In Einzellage	☐	☐	☐	☐	☐	☐
< 100 m vom Waldrand entfernt	☐	☐	☐	☐	☐	☐
Mit Nutzung von Garten/ Schrebergarten	☐	☐	☐	☐	☐	☐

Andere Wohnlage? ☐ Ja ☐ Nein

Wenn ja, welche? ____

**2. Haben Sie jemals in der Nähe von...gewohnt?**				
	Nein	Ja		Weiß nicht
		Bis zu 100 m	Mehr als 100 m	
...einer Wiese/ Weidefläche, Ackerland...	☐	☐	☐	☐
...einem Park oder einer sonstigen Freifläche...	☐	☐	☐	☐
...einem Inlandsgewässer...	☐	☐	☐	☐
...Tierställen, Getreidelagern...	☐	☐	☐	☐
...eigenen Tierställen (z.B. Hasenstall im Garten)...	☐	☐	☐	☐
...ungenutzten Gebäuden...	☐	☐	☐	☐
...einem Waldrand oder kleineren Baumgruppen...	☐	☐	☐	☐

↳ Wenn ja, um was für eine Art von Bäumen handelt/e es sich?

☐Nadelbäume ☐Laubbäume ☐unbekannt

Falls genaue Baumart bekannt, bitte hier eintragen: ____

**3. Wie viele Jahre ihres Lebens hatten Sie folgende Räume/Flächen innerhalb Ihrer Wohnung/Ihres Hauses bzw. auf dem zugehörigen Grundstück zur Verfügung und haben diese genutzt?**						
	Nie	< 1 Jahr	2-5 Jahre	5-10 Jahre	Mehr als 10 Jahre	Immer
Garten	☐	☐	☐	☐	☐	☐
Dachboden	☐	☐	☐	☐	☐	☐
Keller	☐	☐	☐	☐	☐	☐
Geräteschuppen	☐	☐	☐	☐	☐	☐
Garage	☐	☐	☐	☐	☐	☐

**4. Wie häufig haben Sie die folgenden Räume in den vergangenen 10 Jahren gereinigt?**					
	Trifft nicht zu	Gar nicht	1-2 mal	2-5 mal	Mehr als 5 mal
Dachboden	☐	☐	☐	☐	☐
Keller	☐	☐	☐	☐	☐
Geräteschuppen	☐	☐	☐	☐	☐
Garage	☐	☐	☐	☐	☐

Beruf

5a. Sind Sie aktuell im Forstbereich beschäftigt?		
☐Ja<br>➔ weiter mit Frage 7	Wenn ja, wie lange?<br>I__I__I Jahre	☐Nein<br>➔ weiter mit Frage 5b
**5b. Wenn nein, waren sie schon einmal im Forstbereich beschäftigt?**		
☐Ja	Wenn ja, wie lange?<br>I__I__I Jahre	☐Nein

6a. Wenn Sie nicht im Forstbereich arbeiten, welchen Beruf üben Sie aktuell aus?
Bitte hier eintragen: ____
**6b. Wie lange arbeiten Sie schon in diesem Beruf?**
I__I__I Jahre

7a. Haben Sie vor oder neben Ihrer jetzigen Tätigkeit schon mal in einem anderen Beruf bzw. in anderen Berufen gearbeitet?<br><br>(hier bitte auch Landwirtschaft im Nebenerwerb o.ä. angeben)	
☐Ja ➔ weiter mit Frage 7b	☐Nein ➔ weiter mit Frage 8
**7b. Wenn ja, geben Sie bitte an in welchem Beruf und wie lange Sie darin gearbeitet haben?**	
Beruf (bitte eintragen)	Dauer in Jahren
1.	I__I__I Jahre
2.	I__I__I Jahre
3.	I__I__I Jahre

8. Welchen Anteil Ihrer Arbeitszeit verbringen Sie durchschnittlich mit…?<br>(Hinweis: Es sollten insgesamt nicht mehr als 100 % vergeben werden)	
	Anteil in Prozent (%)
reiner Büroarbeit	I__I__I
Wald- und Feldbegehungen	I__I__I
aktiver Waldarbeit	I__I__I
sonstige Arbeiten:<br>(bitte eintragen und bewerten)<br>1. ______________________<br>2. ______________________<br>3. ______________________	I__I__I<br>I__I__I<br>I__I__I

ANGABEN ZUR GESUNDHEIT

9. Rauchen Sie zurzeit – wenn auch nur gelegentlich?	
9a. ☐ Ja	Wie viel rauchen Sie derzeit gewöhnlich?<br><br>I__I__I Anzahl Zigaretten pro Tag
	Falls Sie regelmäßig[1] rauchen:<br><br>Wann haben Sie angefangen, regelmäßig[1] zu rauchen?<br><br>Jahr I__I__I__I__I oder Alter I__I__I
9b. ☐ Nein, ich habe früher regelmäßig[1] geraucht, aber jetzt nicht mehr.	Wie viel haben Sie früher gewöhnlich geraucht?<br><br>I__I__I Anzahl Zigaretten pro Tag
	Wann haben Sie angefangen, regelmäßig[1] zu rauchen?<br><br>Jahr I__I__I__I__I oder Alter I__I__I
	Wann haben Sie aufgehört zu rauchen?<br><br>Jahr I__I__I__I__I oder Alter I__I__I
9c. ☐ Nein, ich habe noch <u>nie</u> regelmäßig[1] geraucht.	

10a. Wurde bei Ihnen jemals eine Infektion oder Erkrankung durch Hantaviren festgestellt und wenn ja, wann?			
☐ Nein<br>➔ weiter mit Frage 11	☐ Ja	In welchem Jahr oder Alter wurde eine Hantavirus-Erkrankung festgestellt?<br><br>Jahr I__I__I__I__I oder Alter I__I__I	☐ Weiß nicht

10b. Wenn Ja, können Sie sich an die Symptome erinnern, die damals bei Ihnen aufgetreten sind?			
	Nein	Ja	Weiß nicht
Abrupt auftretendes hohes Fieber (> 38,5°C)	☐	☐	☐
Starke Kopf-, Rücken-, Muskel- und/oder Gliederschmerzen *(Entsprechendes bitte unterstreichen.)*	☐	☐	☐
Übelkeit, Erbrechen und/oder Durchfall *(Entsprechendes bitte unterstreichen.)*	☐	☐	☐
Schwindel und/oder Kreislaufversagen *(Entsprechendes bitte unterstreichen.)*	☐	☐	☐
Lungenentzündung	☐	☐	☐
Verringerte/ vermehrte Urinausscheidung *(Entsprechendes bitte unterstreichen.)*	☐	☐	☐

[1] **Unter regelmäßig verstehen wir:** 1 Zigarette pro Tag oder mindestens 5 Zigaretten pro Woche oder mindestens 1 Packung Zigaretten pro Monat für mindestens 6 Monate

Fortsetzung zu Frage 10b. ***(Symptome bei Infektion mit Hantaviren)***	Nein	Ja	Weiß nicht
Ungewohnte Blutungsneigung (Nasenbluten, Blut im Stuhl,…)	☐	☐	☐
Nierenfunktionsstörung/ Nierenversagen *(Entsprechendes bitte unterstreichen.)*	☐	☐	☐
Blutwäsche (Dialyse)	☐	☐	☐
Andere Symptome? ☐Ja ☐Nein<br>Wenn Ja, welche? ______________________________			

**11a. Wurde bei Ihnen jemals eine Infektion mit Leptospiren/ Leptospirose festgestellt und wenn ja, wann?**			
☐ Nein ➔ weiter mit Frage 12	☐ Ja	In welchem Jahr oder Alter wurde eine Leptospirose festgestellt?<br>Jahr I__I__I__I__I oder Alter I__I__I	☐ Weiß nicht

**11b. Wenn Ja, können Sie sich an die Symptome erinnern, die damals bei Ihnen aufgetreten sind?**			
	Nein	Ja	Weiß Nicht
Abrupt auftretendes hohes Fieber (> 38,5°C)	☐	☐	☐
Kopf-, Hals-, Bauch-, Muskel-, Gelenk- und/oder Wadenschmerzen *(Entsprechendes bitte unterstreichen.)*	☐	☐	☐
Ausschlag	☐	☐	☐
Blut im Urin	☐	☐	☐
Steifer Nacken	☐	☐	☐
Übelkeit, Erbrechen und/oder Durchfall *(Entsprechendes bitte unterstreichen.)*	☐	☐	☐
Respiratorische Symptome (z.B. Husten, Erkältung,…)	☐	☐	☐
Hirnhautentzündung	☐	☐	☐
Bindehautentzündung (‚rote Augen‘)	☐	☐	☐
Gelbsucht (‚gelbe Augen‘)	☐	☐	☐
Nierenfunktionsstörung/ Nierenversagen *(Entsprechendes bitte unterstreichen.)*	☐	☐	☐
Zweiphasige Erkrankung (Nachdem Sie angefangen haben sich besser zu fühlen, haben Sie einen erneuten Erkrankungsschub mit Fieber bekommen?)	☐	☐	☐
Andere Symptome? ☐Ja ☐Nein<br>Wenn Ja, welche? ______________________________			

FREIZEITVERHALTEN

12. Wie viele Jahre Ihres Lebens hatten Sie oder ein Haushaltsmitglied eines oder mehrere der folgenden Haustiere?

	Nie	< 1 Jahr	2-5 Jahre	6-10 Jahre	Mehr als 10 Jahre	Ein Leben lang
Hund	☐	☐	☐	☐	☐	☐
Katze	☐	☐	☐	☐	☐	☐
Vogel	☐	☐	☐	☐	☐	☐
Fisch	☐	☐	☐	☐	☐	☐
Reptil	☐	☐	☐	☐	☐	☐
Nagetier - Welches? ____________________________	☐	☐	☐	☐	☐	☐
Anderes Haustier - Welches? ____________________________	☐	☐	☐	☐	☐	☐

13. Wie lange gehen Sie *__durchschnittlich unter der Woche (Mo-Fr)__* in Ihrer Freizeit in der Zeit von Frühling bis Herbst folgenden Beschäftigungen nach?
Die Zeitangabe soll für alle Tage zusammen angegeben werden. Die Angabe „2-5 Stunden“ bedeutet also 2-5 Stunden in der Woche von Mo-Fr.

	(Fast) gar nicht	< 1 Stunde	2-5 Stunden	6-10 Stunden	Mehr als 10 Stunden
Aufenthalt im Freien	☐	☐	☐	☐	☐
Aufenthalt im Wald	☐	☐	☐	☐	☐
Arbeit mit Pflanzen und Erde, z.B. Gartenarbeit	☐	☐	☐	☐	☐
Arbeit mit Holz, z.B. Sägen, Stapeln etc.	☐	☐	☐	☐	☐
Kontakt zu Tieren	☐	☐	☐	☐	☐
Arbeit mit Saatgut, Futtermittel, Dünger o.ä.	☐	☐	☐	☐	☐

**14. Wie lange gehen Sie *<u>durchschnittlich am Wochenende (Sa-So)</u>* in Ihrer Freizeit in der Zeit von Frühling bis Herbst folgenden Beschäftigungen nach?**<br>*Die Zeitangabe soll für das gesamte Wochenende angegeben werden. Die Angabe „2-5 Stunden" bedeutet also 2-5 Stunden an einem Wochenende (Sa-So).*					
	(Fast) gar nicht	< 1 Stunde	2-5 Stunden	6-10 Stunden	Mehr als 10 Stunden
Aufenthalt im Freien	☐	☐	☐	☐	☐
Aufenthalt im Wald	☐	☐	☐	☐	☐
Arbeit mit Pflanzen und Erde, z.B. Gartenarbeit	☐	☐	☐	☐	☐
Arbeit mit Holz, z.B. Sägen, Stapeln etc.	☐	☐	☐	☐	☐
Kontakt zu Tieren	☐	☐	☐	☐	☐
Arbeit mit Saatgut, Futtermittel, Dünger o.ä.	☐	☐	☐	☐	☐

**15. Wie häufig unternehmen Sie in Ihrer Freizeit folgende Aktivitäten „im Grünen"?**			
	Nie	Selten	Häufig
Spazieren gehen, Wandern, Joggen	☐	☐	☐
Jagen, Angeln	☐	☐	☐
Arbeit im Garten/ Schrebergarten	☐	☐	☐
Sonstige? ☐Ja ☐Nein<br>Welche? ____	☐	☐	☐

**16. Wie häufig gehen Sie in Ihrer Freizeit folgenden Wassersportarten nach?**			
	Nie	Selten	Häufig
Surfen/ Kiten	☐	☐	☐
Segeln	☐	☐	☐
Tauchen	☐	☐	☐
Kanu/ Kajak/ Rafting	☐	☐	☐
Baden in Inlandsgewässern (z.B. See, Bach oder Teich)	☐	☐	☐
Sonstige? ☐Ja ☐Nein<br>Welche? ____	☐	☐	☐

KONTAKT ZU NAGETIEREN

17. Wie häufig haben Sie in den vergangenen 10 Jahren in Ihrem Wohnumfeld/ Arbeitsumfeld Anzeichen für Nagetiere entdeckt?					
	Gar nicht	1-2 mal	3-5 mal	6-20 mal	Mehr als 20 mal
Kot	☐	☐	☐	☐	☐
Nagespuren	☐	☐	☐	☐	☐
Schmierspuren	☐	☐	☐	☐	☐
Nester, Bauten	☐	☐	☐	☐	☐
Tote Tiere	☐	☐	☐	☐	☐
Lebende Tiere	☐	☐	☐	☐	☐
Veränderter Geruch	☐	☐	☐	☐	☐

18. Wie häufig wurden bzw. haben Sie in den vergangenen 10 Jahren…?					
	Gar nicht	1-2 mal	3-5 mal	6-20 mal	Mehr als 20 mal
von Nagetieren gebissen	☐	☐	☐	☐	☐
lebendige Nagetiere berührt	☐	☐	☐	☐	☐
tote Nagetiere berührt	☐	☐	☐	☐	☐
Tierställe gereinigt	☐	☐	☐	☐	☐
Lagerräume, Garagen, Dachböden o.ä. ausgefegt oder aufgeräumt	☐	☐	☐	☐	☐

WISSEN ÜBER ERKRANKUNGEN

19. Haben Sie bereits von Hantaviren gehört? Wenn Ja, wodurch und wie oft?					
	Nie	Ca. einmal	2-10 mal	Mehr als 10 mal	Immer wieder
Bekannte, Kollegen, Nachbarn	☐	☐	☐	☐	☐
Lokalzeitung, Zeitschrift	☐	☐	☐	☐	☐
Fernsehen, Radio	☐	☐	☐	☐	☐
Internet, Soziale Medien (WhatsApp, Facebook,…)	☐	☐	☐	☐	☐
Information durch Gemeinde, Landkreis, Gesundheitsamt	☐	☐	☐	☐	☐
Information durch Arbeitgeber	☐	☐	☐	☐	☐

20. Haben Sie bereits von Leptospiren bzw. Leptospirose gehört? Wenn Ja, wodurch und wie oft?					
	Nie	Ca. einmal	2-10 mal	Mehr als 10 mal	Immer wieder
Bekannte, Kollegen, Nachbarn	☐	☐	☐	☐	☐
Lokalzeitung, Zeitschrift	☐	☐	☐	☐	☐
Fernsehen, Radio	☐	☐	☐	☐	☐
Internet, Soziale Medien (WhatsApp, Facebook,…)	☐	☐	☐	☐	☐
Information durch Gemeinde, Landkreis, Gesundheitsamt	☐	☐	☐	☐	☐
Information durch Arbeitgeber	☐	☐	☐	☐	☐

PRÄVENTIONSMAßNAHMEN

21. Haben Sie in der Vergangenheit bewusst Schutzmaßnahmen angewendet, um sich vor Infektionen zu schützen, die durch Nagetiere oder deren Ausscheidungen übertragen werden können?					
	Trifft nicht zu	Nie	Manchmal	Immer	Nur wenn ich vor Kurzem davon gehört hab
Mund- und Nasenschutz beim Ausfegen von Lagerräumen oder Arbeiten mit Holzstapeln oder Reinigen von Ställen	☐	☐	☐	☐	☐
Schutzhandschuhe vor dem Berühren von Nagetieren	☐	☐	☐	☐	☐
Händewaschen nach dem Berühren von Nagetieren	☐	☐	☐	☐	☐
Schutzhandschuhe bei Tätigkeiten mit Pflanzen und Erde	☐	☐	☐	☐	☐

WEITERE ANGABEN ZU IHRER PERSON

22. Welchen höchsten allgemeinbildenden Schulabschluss haben Sie?	
Schüler/in, Besuch einer allgemeinbildenden Vollzeitschule	☐
Von der Schule abgegangen ohne Schulabschluss	☐
Hauptschulabschluss (Volksschulabschluss) oder gleichwertiger Abschluss	☐
Polytechnische Oberschule der DDR mit Abschluss der 8. oder 9. Klasse	☐
Realschulabschluss (Mittlere Reife) oder gleichwertiger Abschluss	☐
Polytechnische Oberschule der DDR mit Abschluss der 10. Klasse	☐
Fachhochschulreife	☐
Abitur/ Allgemeine oder fachgebundene Hochschulreife (Gymnasium bzw. EOS, auch EOS mit Lehre)	☐
Einen anderen Schulabschluss? ☐Ja ☐Nein<br>Welchen?<br>______________________________	☐

VIELEN DANK!
